# An Integrated Study on the Differential Expression of the *FOX* Gene Family in Cancer and Their Response to Chemotherapy Drugs

**DOI:** 10.3390/genes13101754

**Published:** 2022-09-28

**Authors:** Haimeng Yin, Xing Fan, Yanqiao Zhang, Nan Zhao, Xiaoyi Zhao, Kehan Yin, Yali Zhang

**Affiliations:** School of Medicine, Nantong University, Nantong 226001, China

**Keywords:** Forkhead-box family genes, overlapping expression, cancer diseases, anticancer drugs

## Abstract

The Forkhead-box (*FOX*) transcription factors, as one of the largest gene families in humans, play key roles in cancer. Although studies have suggested that several *FOX* transcription factors have a significant impact on cancer, the functions of most of the *FOX* genes in cancer remain elusive. In the study, the expression of 43 *FOX* genes in 63 kinds of cancer diseases (including many subtypes of same cancer) and in response to 60 chemical substances was obtained from the Gene Expression Atlas database of the European Bioinformatics Institute. Based on the high degree of overlap in *FOXO* family members differentially expressed in various cancers and their particular responses to chemotherapeutic drugs, our data disclosed the *FOX* genes that played an important role in the development and progression of cancer. More importantly, we predicted the role of one or several combinatorial *FOX* genes in the diagnosis and prognostic assessment of a specific cancer and evaluated the potential of a certain anticancer drug therapy for this type of cancer by integrating patterns of *FOX* genes expression with anticancer drugs sensitivity.

## 1. Introduction

The Forkhead-box (*FOX*) transcription factors contain a highly conserved winged DNA-binding domain, which is composed of about 100 similar amino acids [1]. Genome-based bioinformatics analyses have shown that *FOX* genes were classified into 23 subfamilies [2,3,4]. In humans, there are more than 50 members from *FOXA* to *FOXS* subfamilies in the *FOX* superfamily [2,3,4]. *FOX* transcription factors have been demonstrated to regulate diverse biological processes related to cell differentiation, cell cycle control, embryonic development, metabolism, longevity, etc. [3]. Mutations of many *FOX* genes have been associated with human genetic diseases in different tissues, such as the brain, heart, liver, kidney, lung, breast, prostate, pancreas, vascular tissue, and immune cells [3,4,5]. Most importantly, *FOX* family genes, such as *FOXA1*, *FOXC2*, *FOXE1*, *FOXF1*, *FOXM1*, *FOXO1*, *FOXO3, FOXO4*, *FOXO6*, *FOXP1*, *FOXP3*, *FOXQ1*, and *FOXR1*, have been documented to play key roles in cancer by acting as tumor suppressors and/or oncogenes genes [3,4,6,7].

Although the function of several *FOX* transcription factors in cancers has been characterized, the functions of most *FOX* genes in cancer, especially in the subtypes of same cancer and in the response to anticancer drugs, are still unknown. In the study, we analyzed that the expression of 43 *FOX* genes in 63 kinds of cancer diseases (including many subtypes of the same cancer) and in response to 60 chemical substances. By integrating patterns of *FOX* genes expression with anticancer drugs sensitivity, we speculated that six *FOX* genes, including *FOXO3*, *FOXO1*, *FOXN3*, *FOXK2*, *FOXN2*, and *FOXJ3*, might be important for the development and treatment of a wide range of cancers. Furthermore, we revealed the value of the combination of several *FOX* genes in the diagnosis of the corresponding cancer and assessed the novel therapeutic potential of anticancer drugs by targeting these genes. Our study aimed to put up new clues for the further functional study of *FOX* genes in the occurrence and therapy of cancers.

## 2. Materials and Methods

### 2.1. Gene Correlation Analysis in Gene Expression Atlas

The differential expression of human *FOX* genes in different diseases and response to different chemical substances were obtained from the Gene Expression Atlas database of the European Bioinformatics Institute (http://www.ebi.ac.uk/gxa, accessed on 20 May 2021) [8]. The Gene Expression Atlas is a comprehensive *p*-value database, based on many statistical methods and various independent studies, that provides information on the expression of genes or proteins in different species and conditions, including the cell types, different tissues, diseases, and chemical substances. An interface enables us to search for the expression of genes (i) by *FOX* genes (gene/gene properties); (ii) by Homo sapiens (organism); and (iii) by cancer diseases or chemical treatment response (biological conditions). Using the condition of a *p*-value < 0.05 for querying, among all the *FOX* genes in humans, the differential expression of 43 *FOX* genes in different cancer diseases and response to different chemical substances were obtained, except *FOXD4L2-FOXD4L6*, because of their lack of expression.

To reflect the significant difference level, a *p*-value < 0.0001 of downregulated genes was set as the number 1 and is shown in deep green; 0.0001 < *p*-value < 0.05 of downregulated genes was set as the number 2 and is shown in light green; the *p*-value > 0.05 indicates a lack of differential expression, was set as the number 3, and is shown in black; 0.0001 < *p*-value < 0.05 of upregulated genes was set as the number 4 and is shown in light red; and a *p*-value < 0.0001 of upregulated genes was set as the number 5 and is shown in deep red. Moreover, the inverted *p*-values and their corresponding *FOX* genes are shown in the figures.

### 2.2. Other Gene Correlation Analysis Methods

The Gene Expression Profiling Interactive Analysis (GEPIA2) (http://gepia2.cancer-pku.cn/#index, accessed on 22 August 2022) online database was used to testify the notably related genes between clinically visible tumors and normal tissues [9]. GEPIA2 is an online commonly tool to calculate survival analysis automatically by analyzing the RNA-seq results. In addition, the University of Alabama at Birmingham Cancer data analysis portal (UALCAN) (http://ualcan.path.uab.edu/index.html, accessed on 2 May 2022) was also applied to the prognosis survival analysis [10]. The representative immunohistochemistry plots of the certain molecular was detected from The Human Protein Atlas (HPA) (https://www.proteinatlas.org/, accessed on 2 May 2022) website [11]. To depict the genetic characteristics of cancer cells, several data were obtained from a comprehensive interactive web resource—Cancer Cell Line Encyclopedia (CCLE) (https://portals.broadinstitute.org/ccle/data, accessed on 10 October 2021) [12]. STRING (https://string-db.org/, accessed on 10 October 2021) is a database of known and predicted functional protein-protein interaction (PPI) networks [13]. The TCGA database (https://portal.gdc.cancer.gov/, accessed on 4 September 2022) was used to obtain clinical information on breast cancer patients and the relating RNA-Seq and gene mutation data [14]. The co-occurrence and co-exclusion analysis was performed by maftools package in R software [15].

## 3. Results

### 3.1. Identification of FOX Genes That Play Important Roles in Various Types of Cancer

As shown in Figure S1, the differential expression of 43 *FOX* genes in 63 kinds of cancers (including many subtypes of same cancer), such as leukemia, lymphoma, brain tumors, prostate cancer, breast carcinoma, kidney carcinoma, lung carcinoma, and pancreatic cancer, and might imply that these *FOX* genes have pleiotropic effect in cancers. The expressions of FOXF1, FOXJ2, FOXJ3, FOXK2, FOXM1, FOXN2, FOXN3, FOXO1, FOXO3, FOXO4, and FOXP1 were markedly different in multiple cancers. It is worth noting that *FOX* genes showed overlapping expression patterns in subtypes of the same cancer.

#### 3.1.1. Expression Profile of *FOX* Genes in Leukemia and Identification of Several *FOX* Genes with High Expression in AML

**Figure 1:**
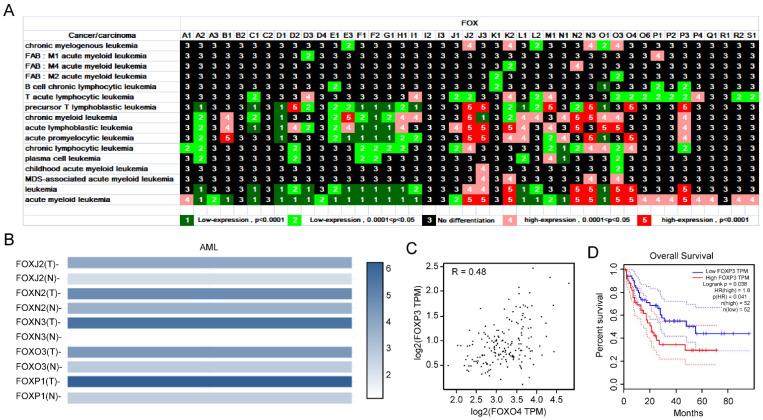
Differential expression of *FOX* genes in leukemia (**A**). (**B**) The expression of *FOXJ2*, *FOXN2*, *FOXN3*, *FOXO3*, and *FOXP1* in AML and normal samples. (**C**) The Pearson Correlation analysis of *FOXO4* and *FOXP3* expression in AML. (**D**) Survival analysis curves of *FOXP3* in AML.

Leukemia, a common tumor disease of the blood or bone marrow, characterized by the presence of immature leukocytes, is a major cause of death from cancer [16,17]. Leukemia can be divided into different subtypes, including chronic myeloid leukemia (CML), chronic lymphocytic leukemia (CLL), acute myeloid leukemia (AML), and acute lymphoblastic leukemia (ALL) [18,19,20]. The expression levels of FOXA2, FOXC1, FOXD1, FOXE1, FOXF1, FOXF2, and FOXG1 were downregulated in all types of leukemia, while those of FOXJ2, FOXJ3, FOXM1, FOXN2, FOXN3, FOXO3, FOXO4, and FOXP3 were upregulated in at least four subtypes of leukemia (Figure 1A). In addition, FOXK2, FOXL1, FOXO1, and FOXP3 were differentially expressed in at least six subtypes of leukemia (Figure 1A). In parallel, we compared the mRNA expression of FOXJ2, FOXN2, FOXN3, FOXO3, and FOXP1 between AML and normal samples by using the GEPIA2 database. Results indicated that these five genes were all upregulated in AML samples (Figure 1B). Given that FOXO4 and FOXP3 were highly expressed in combination in precursor T lymphoblastic leukemia, acute promyelocytic leukemia, leukemia, and acute myeloid leukemia, we explored the correlation of these two genes in the GEPIA2 database and found that they were positively correlated in AML (Figure 1C). AML patients with high FOXP3 expression tend to have a poor prognosis as well (Figure 1D).

#### 3.1.2. Importance of *FOXO1* and *FOXO4* from the Expression Profile of *FOX* Genes in Lymphoma

**Figure 2:**
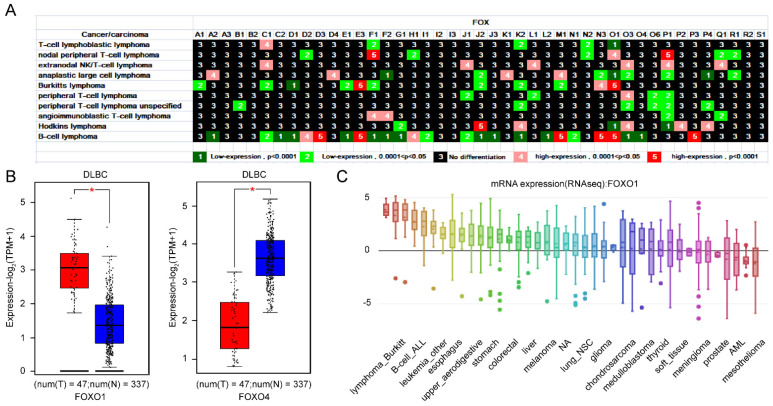
Differential expression of *FOX* genes in lymphoma (**A**). (**B**) The expression level of *FOXO1* and *FOXO4* between DLBC and normal tissues. (**C**) Characterization of *FOXO1* in cancer cell lines. *p* values less than 0.05 were considered to be statistically significant, * *p* < 0.05.

Lymphoma, as a type of neoplastic disease, starts in lymphocytes or white blood cells and can spread quickly from the lymph nodes to other body parts through the lymphatic system [21,22]. Hodgkin lymphoma is believed to involve lymphocytes, eosinophilic granulocytes, plasma cells, and specific Reed–Sternberg cells in tumor tissues. Non-Hodgkin lymphoma, the most common type, comprises many subtypes, such as T-cell, B-cell, and NK lymphoma [21,22]. Ten subtypes of lymphoma were investigated in our study (Figure 2A). FOXF1, FOXK2, FOXO1, FOXO3, and FOXP1 were differentially expressed in at least five subtypes of lymphoma. Twenty-five *FOX* genes were differentially expressed in B-cell lymphoma (Figure 2A). Interestingly, both belong to the FOXO family, and compared with normal samples, FOXO1 was highly expressed in lymphoid neoplasm diffuse large B-cell lymphoma (DLBC); however, FOXO4 was down expression in DLBC (Figure 2B). Particularly, FOXO4 is downregulated only in DLBC, but not in other types of lymphomas (Figure 2A). In addition, high expression of FOXO1 was observed in lymphoma cell lines (Figure 2C).

#### 3.1.3. Upregulation of *FOXD1* Based on TP53 Mutation Is a Poor Prognosis Factor for LGG Patients

**Figure 3:**
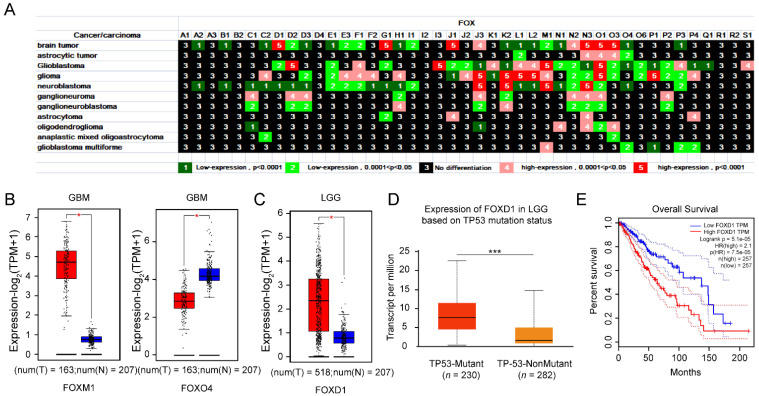
Differential expression of *FOX* genes in nervous system tumors (**A**). (**B**) The expression of *FOXM1* and *FOXO4* in GBM and normal tissues through GEPIA2. (**C**) The differential expression of *FOXD1* between LGG and Normal groups. (**D**) The box plot of *FOXD1* based on TP53 mutation statues in UALCAN-LGG dataset. (**E**) The survival curve of high and low expression groups for *FOXD1* in LGG patients. *p* values less than 0.05 were considered to be statistically significant, * *p* < 0.05, *** *p* < 0.001.

Central nervous system tumors are types of high mortality in nervous system diseases [23]. There were 11 kinds of nervous system tumors in our study (Figure 3A). In total, 25, 27, 24, and 25 *FOX* genes were differentially expressed in brain tumors (8 upregulated and 17 downregulated), glioblastoma (10 upregulated and 17 downregulated), glioma (14 upregulated and 10 downregulated), and neuroblastoma (4 upregulated and 21 downregulated), respectively. FOXD2, FOXD3, FOXH1, FOXJ3, FOXK2, FOXL1, FOXM1, FOXN2, FOXN3, FOXO1, FOXO3, and FOXP3 were differentially expressed in at least five types of nervous system tumors. The same was true for the upregulation of FOXM1 and downregulation of FOXO4 in glioblastoma multiforme (GBM) (Figure 3B). Besides, the FOXD1 expression in the bran lower-grade glioma (LGG) group was higher than that in the control group (Figure 3C). It is well known that TP53 is a tumor suppresser, and according to the ALCAN-LGG dataset, the expression level of FOXD1 was high in the TP53 mutant group (Figure 3D). Furthermore, the high expression of FOXD1 conferred a poor prognosis of survival in LGG patients (Figure 3E).

#### 3.1.4. The Importance of *FOXA1* in Prostate as Well as Its Interaction with GATA3 and PGR

**Figure 4:**
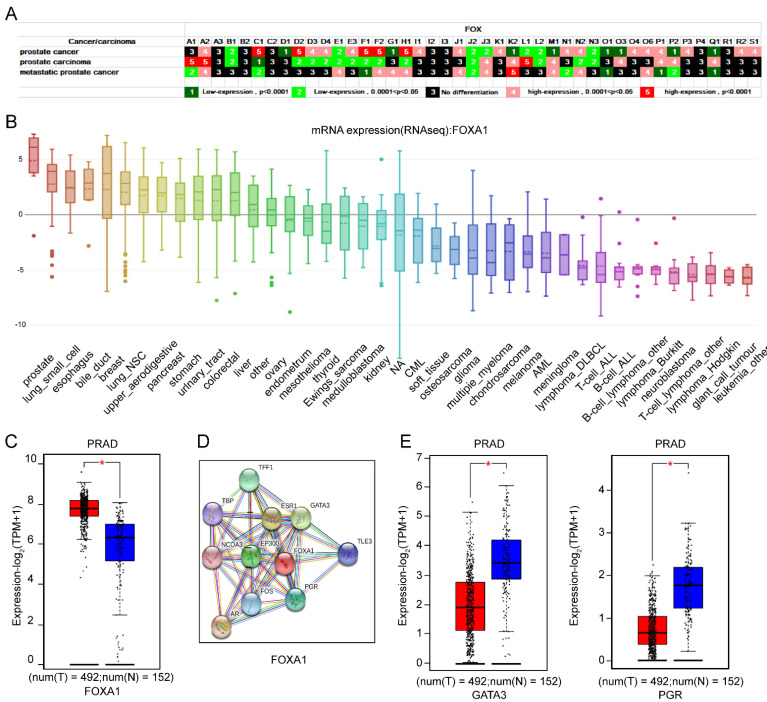
Differential expression of *FOX* genes in prostate cancer (**A**). (**B**) The expression of *FOXA1* in different cell lines. (**C**)The Differential expression analysis of *FOXA1* was carried out between PRAD and normal tissues. (**D**) The respective PPI network of *FOXA1*. (**E**) Difference of GATA3 and PGR expression between PRAD and normal tissues. *p* values less than 0.05 were considered to be statistically significant, * *p* < 0.05.

An investigative report in 2012 showed that 1.1 million men, accounting for more than 15% of the cancers diagnosed in men, were diagnosed with prostate cancer [24]. The FOX transcription factors have been regarded as direct or indirect factors for predicting, monitoring, and curing prostate cancer [25]. As shown in Figure 4A, almost all of the *FOX* genes were differentially expressed in prostate cancer, prostate carcinoma, and metastatic prostate cancer, except FOXA3, FOXB2, FOXC2, FOXI2, FOXI3, FOXP4, and FOXR1. The FOXA1 was identified as a recurrent mutation in prostate carcinoma by exome sequencing [26]. The finding of Jin’s group illustrated that recurrent mutation of FOXA1 lead to prostate carcinoma progress [27]. As shown in Figure 4B,C, FOXA1 is highly expressed in prostate cancer cells and prostate adenocarcinoma (PRAD). To explore novel molecules that interacted with FOXA1 in PRAD, we utilized the existing interactive gene network from the STRING software (Figure 4D). Surprisingly, although GATA3 and PGR both closely interacted with FOXA1, they were downregulated in PRAD (Figure 4E). Recent studies have revealed that the GATA3/miR−503/ZNF217 axis influenced the epithelial-mesenchymal transition process in PRAD [28], and GATA3-dependent mitotic spindle orientation was crucial for prostate lineage specification [29]. The roles of PGR-related genes in prostate cancer tissues have also been summarized [30]. These results indicated that the downregulation of GATA3 and PGR is a critical determinant of the progression in PRAD, possibly modulated by FOXA1. The underlying mechanism is under further investigation.

#### 3.1.5. Multiple Roles of *FOX* Gene in Breast Cancer and Identification of Several *FOX* Genes with High Expression in TNBC

**Figure 5:**
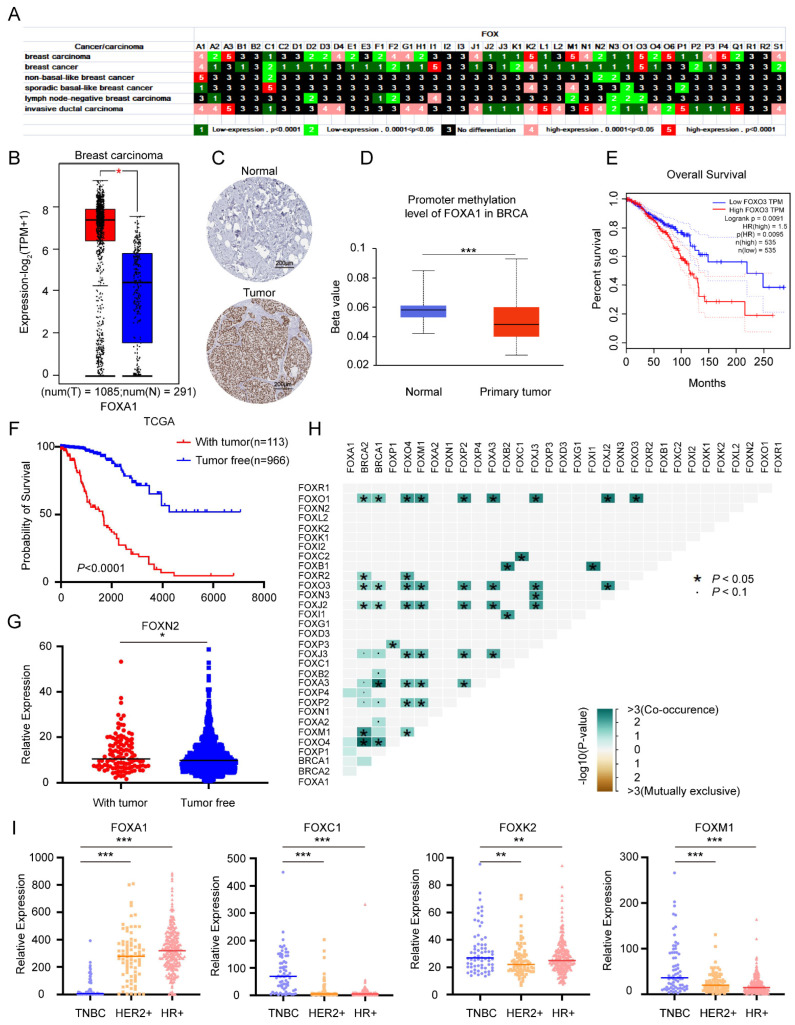
Expression profile of *FOX* genes in breast cancer (**A**). (**B**) The representative expression of *FOXA1* in the GEPIA2 dataset. (**C**) Differential expression of *FOXA1* in normal breast tissue and breast cancer tissue was retrieved from the HPA database. (**D**) Box plot of *FOXA1* promoter methylation expression level in UALCAN-BRCA dataset. (**E**) The survival curve of BRCA patients with different *FOXO3* expression levels. (**F**) A prognostic analysis based on the TCGA database of breast carcinoma patients with and without relapsed tumor groups. (**G**) The differential expression of *FOXN2* in breast carcinoma patients between “with tumor” and “tumor free” groups. (**H**) The co-occurrence and co-exclusion analysis of the *FOX* family and *BRCA1/2* mutated genes. (**I**) The relative expression of *FOXA1, FOXC1, FOXK2*, and *FOXM1* in three subtypes of breast cancer (TNBC: Triple-Negative Breast Cancer; HER2+: human epidermal growth factor receptor 2 positive; HR+: estrogen and progesterone receptors positive). *p* values less than 0.05 were considered to be statistically significant, * *p* < 0.05, ** *p* < 0.01, *** *p* < 0.001.

Breast cancer, as a high morbidity and mortality kind of cancer in women, is expected to increase significantly in the future [31,32]. The expression of FOXO3a is a favorable prognostic marker in breast cancer [33]. FOXA1 overexpression is involved in estrogen receptor-positive breast cancer [34]. Compared with its expression in normal breast tissue, FOXM1 has shown a different expression pattern in breast neoplasms [35]. As shown in Figure 5A, among the 6 kinds of breast carcinoma/cancers, 32 (14 upregulated and 18 downregulated), 31 (4 upregulated and 27 downregulated), and 28 (17 upregulated and 11 downregulated) *FOX* genes were differentially expressed in breast carcinoma, breast cancer, and invasive ductal carcinoma, respectively. Data-mining results implied that FOXA1 expressed at a high level in breast carcinoma (Figure 5B). According to the HPA dataset, the positive rate of FOXA1 expression was high in tumor tissues, whereas all the normal breast tissues were not stained (Figure 5C). It is documented that promoter hypermethylation is associated with gene silencing. Based on the predicted results from the UALCAN database, promoter methylation levels of FOXA1 were lower in breast carcinoma compared to normal tissues (Figure 5D). These results highlight the crucial role that FOXA1 upregulation play in breast carcinoma development and progression. In addition, FOXO3 exerted a higher expression level in breast cancer (Figure 5A) and was significantly correlated with a poor prognosis (Figure 5E). Based on the “person neoplasm cancer status” in the TCGA database, we regarded the “with tumor” group as a recurrence group and the “tumor free” group as a non-recurrence group. The prognosis for breast cancer patients with relapsed tumors is poor (Figure 5F). In addition, FOXN2 expression was elevated slightly in the recurrence group, compared to the non-recurrence group (Figure 5G). Since the prevalence of breast cancer increases drastically in individuals with BRCA1/2 mutation [36], we performed a collinear analysis of the FOX family and BRCA1/2 gene mutations in breast cancer patients based on data from TCGA. The results suggested that mutations in FOXO1, FOXO3, FOXJ2, and FOXO4 are associated with mutations in BRCA1/2 (Figure 5H). Taken together, *FOX* genes are widely involved in breast cancer including occurrence, prognosis, and recurrence of breast cancer.

Triple-negative breast cancer (TNBC), the most aggressive subtype of breast cancer, refers to a broad category of breast cancer that tests negative for estrogen receptors (ER), progesterone receptors (PRs), and human epidermal growth factor receptor 2 (HER2). Despite recent progress on the underlying tumor biology, TNBC has a poor prognosis and limited molecular-targeted treatment options [37]. According to the gene expression profile, we divided the breast cancer data obtained from the TCGA database into three types: TNBC, HER2 positive (HER2+) breast cancer, and hormone receptors-positive (HR+) breast cancer to further explore the expression differences of *FOX* genes. As shown in Figure 5I, compared with HER2+ and HR+ breast cancers, FOXA1 was lowly expressed in TBNC, while FOXC1, FOXK2, and FOXM1 were highly expressed.

#### 3.1.6. The Importance of *FOXO1* and *FOXO4* in KIRC from Expression, Prognosis, and Correlation Analysis

**Figure 6:**
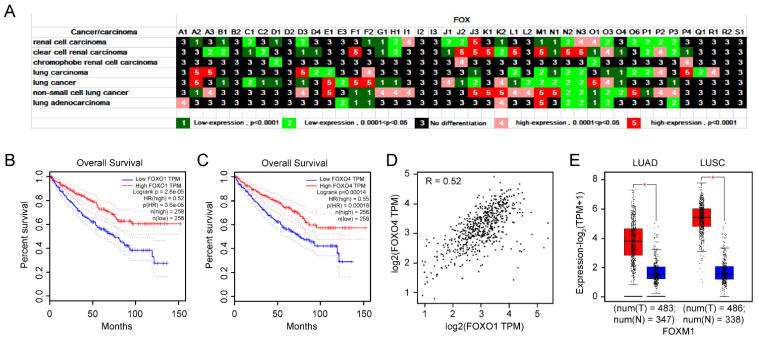
Differential expression of *FOX* genes in renal cell carcinoma and lung cancer (**A**). (**B**,**C**) Effects of the respective expression level of *FOXO1* and *FOXO4* on the survival rate of KIRC patients. (**D**) Pearson correlation coefficient analysis of *FOXO1* and *FOXO4* in KIRC tumor and normal tissues. (**E**) The differential expression of *FOXM1* in LUAD, LUSC, and normal tissues. *p* values less than 0.05 were considered to be statistically significant, * *p* < 0.05.

About 431,288 persons were diagnosed with kidney carcinoma in 2020, accounting for 2.2% of the total number of all cancers diagnosed globally [38]. Most of the *FOX* genes were differentially expressed in renal cell carcinoma (23 *FOX* genes) and clear cell renal carcinoma (26 *FOX* genes), while only 4 *FOX* genes were differentially expressed in chromophobe renal cell carcinoma (Figure 6A). The disordered expression of FOXD4L2, FOXK2, and FOXL1 could be prognostic markers for kidney renal clear cell carcinoma (KIRC) patients [39]. As can be seen from Figure 6A, FOXO1 and FOXO4 exerted a lower expression level in KIRC. By querying the GEPIA2 database, high expression of FOXO1 and FOXO4 were associated with a good prognosis of KIRC (Figure 6B,C). Simultaneously, positive correlation between FOXO1 and FOXO4 expression was observed in Figure 6D.

Lung cancer is one of the main cancers to cause people death in the world [40]. A total of 36 *FOX* genes were differentially expressed in lung carcinoma, lung cancer, non-small cell lung cancer, and lung adenocarcinoma. Among the *FOX* genes, FOXA2, FOXD3, FOXE1, FOXF1, FOXF2, FOXJ3, FOXK2, FOXL1, FOXM1, FOXN1, FOXN2, FOXN3, FOXO1, FOXO3, FOXO4, and FOXP3 were differentially expressed in at least three kinds of lung cancer (Figure 6A). Compared to normal tissues, either in lung adenocarcinoma (LUAD) or lung squamous cell carcinoma (LUSC), the expression of FOXM1 is at a higher level (Figure 6E).

#### 3.1.7. The Importance of *FOXM1* and *FOXP1* in PAAD Is Underlined by Their Expression and Prognosis

**Figure 7:**
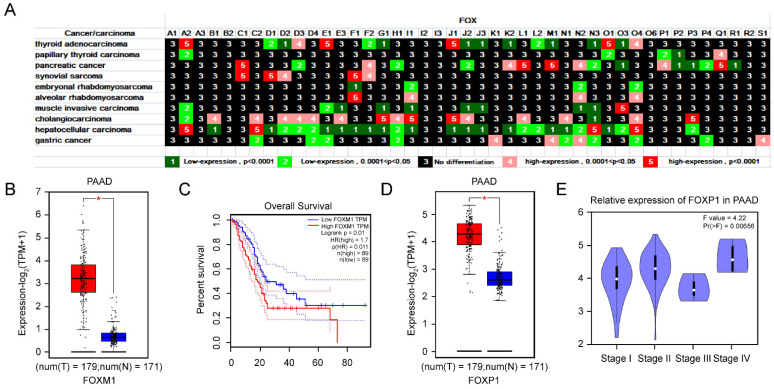
Differential expression of *FOX* genes in other cancers (**A**). (**B**) GEPIA2 data of *FOXM1* expression in PAAD and normal samples. (**C**) The survival curve of PAAD patients with different *FOXM1* expression level. (**D**) Difference of *FOXP1* expression between PAAD and normal tissues. (**E**) Violin plots of the expression of FOXP1 with tumor stage in PAAD. *p* values less than 0.05 were considered to be statistically significant, * *p* < 0.05.

*FOX* genes were differentially expressed in other cancers, such as thyroid adenocarcinoma, papillary thyroid carcinoma, pancreatic cancer, synovial sarcoma, embryonal rhabdomyosarcoma, alveolar rhabdomyosarcoma, muscle-invasive carcinoma, cholangiocarcinoma, hepatocellular carcinoma, and gastric cancer (Figure 7A). Through GEPIA, FOXM1 exerted a higher expression in pancreatic adenocarcinoma (PAAD), and corresponded with a poor prognosis (Figure 7B,C). Notably, FOXP1 was highly expressed in PAAD (Figure 7D), with different expression levels in different stages (Figure 7E).

### 3.2. Integrative Analysis of FOX Genes Differential Expression and Response to Chemicals

#### 3.2.1. Screening for *FOX* Genes Which Are Important for the Progression and Treatment of a Wide Range of Cancers

**Figure 8:**
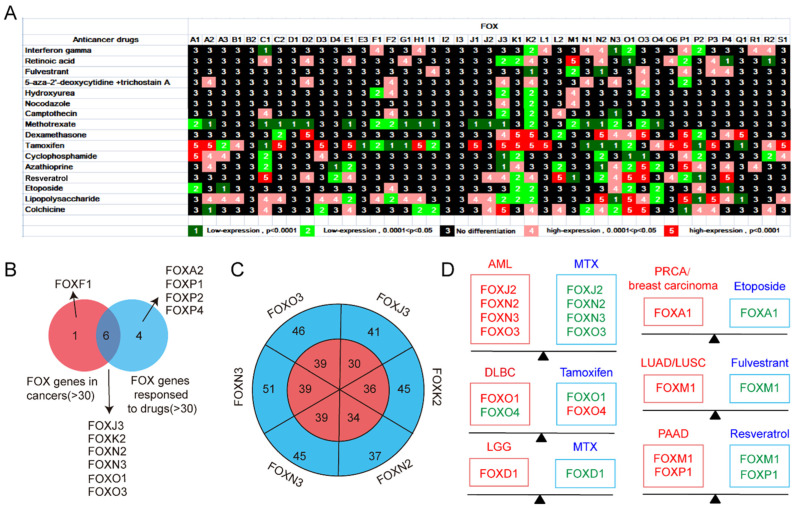
Differential expression of *FOX* genes response to anticancer drugs (**A**). (**B**) Statistics of *FOX* genes differentially expressed in various tumors (>30) and sensitive to multiple chemotherapeutic agents (>30). (**C**) The corresponding numbers of tumors (in the red circle) and chemical substances (in the blue circle) of six *FOX* genes. (**D**) The potential therapeutic roles of anticancer drugs to certain tumors due to opposing regulatory effects on the same *FOX* genes.

The differential expression of human *FOX* genes in response to different chemical substances was also analyzed. A total of 16 kinds of anticancer drugs, including interferon γ, fulvestrant, camptothecin, tamoxifen, lipopolysaccharide, and colchicine, were analyzed, as shown in Figure 8A. The results showed that *FOX* genes were also differentially expressed in response to 60 different chemical substances (Figure S2), including some anticancer drugs.

We found that almost the majority of *FOX* genes differentially expressed in multiple cancers (>30) showed sensitivity to multiple chemotherapeutic agents (>30), suggesting that these *FOX* genes, including FOXJ3, FOXK2, FOXN2, FOXN3, FOXO1, and FOXO3, play an important role in the initiation and progression of cancers and could serve as important therapeutic targets for cancers (Figure 8B,C).

#### 3.2.2. Evaluating the Potential of Anticancer Drug Therapy for a Specific Cancer

Based on the above research results, we wondered whether we could actively explore new anticancer effects of known anticancer drugs through the differential expression of *FOX* genes in tumors and the different responses to drugs. In order to find novel matching anticancer drugs for the above-mentioned malignant tumors, we analyzed the *FOX* genes in Figure S1 and Figure 8A. As shown in Figure 8D, the expression of FOXJ2, FOXN2, FOXN3, and FOXO3, which were originally highly expressed in AML, was suppressed after the stimulation of MTX. The treatment of Tamoxifen could cause the decrease of FOXO1 and the increase of FOXO4, which were differently expressed in DLBC. In similar, MTX, Etoposide, Fulvestrant, and Resveratrol could suppress the expression of FOXD1, FOXA1, FOXM1, and FOXP1, respectively, which exhibited oncogenic potential in LGG, PRCA/breast cancer, LUAD/LUSC, and PAAD. More research is needed to explore and identify the new applications of known anticancer drugs.

## 4. Discussion

The *FOX* transcription factors are a family of evolutionarily conserved transcriptional factors characterized by a winged helix/forkhead DNA-binding domain, which has a consensus sequence GTAAATAAA [41]. Apart from its common role in physiological processes, dysregulation of *FOX* transcription factors has been reported to be closely associated with tumor initiation and development, raising the possibility that *FOX* transcriptional factors combination could serve as effective diagnostic markers and novel therapeutic targets for cancer. Moreover, chemical substances targeting specific *FOX* genes could be considered as effective curative options.

In this study, we conducted a differential expression analysis of 43 *FOX* genes in 16 leukemia subtypes. Among the 43 *FOX* genes, we found that *FOXA2*, *FOXC1*, *FOXD1*, *FOXE1*, *FOXF1*, *FOXF2*, *FOXG1*, *FOXJ2*, *FOXJ3*, *FOXM1*, *FOXN2*, *FOXN3*, *FOXO3*, *FOXO4*, and *FOXP3* were differentially expressed in a large number of leukemias with significant changes, suggesting that these genes play an important role in the pathogenesis of leukemia (Figure 1A). Over the past decade, AML has been the most common form of acute leukemia among adults, with little advancement in cure rates. The highly expressed *FOXJ2*, *FOXN2*, *FOXN3*, *FOXO3*, and *FOXP1* were observed in AML (Figure 1B). Among all the regulated *FOX* genes in leukemia, the high expression of *FOXO4* and *FOXP3* in multiple leukemia types and their correlation, as well as the prognostic value of *FOXP3* in AML, suggest the importance of *FOXO4* and *FOXP3* in leukemia, especially AML, and the potential of their combination as biomarkers for AML diagnosis and drug sensitivity.

Consistent with our finding, several lines of evidence have recently highlighted the roles of *FOXN3* [42], *FOXO3* [43], *FOXO4* [16], and *FOXP3* [44] in leukemia. In this light, based on the fully elucidation of pathogenic mechanisms of these genes, the identification of new medicine targeting these genes could be considered as a novel clue provided for effective systemic therapy. As illustrated in Figure 8A, the expression of *FOXJ2*, *FOXN2*, *FOXN3*, *FOXO3*, and *FOXO4* are decreased after Methotrexate (MTX) stimulation. Zhao et al. demonstrated the potential clinical value of MTX by an aptamer-drug conjugation, which resulted in the apoptosis of AML cells [45]. It is reasonable to speculate that the combination therapy with MTX may be an attractive way to give a possibility in improving the therapeutic efficacy of AML.

It is reported that *FOXO1* confers long-term maintenance of the dark zone proliferation program and can acted as a pharmacologically target in adult Burkitt Lymphoma [46]. This report is in line with our results that *FOXO1* was not only differentially expressed in six types of lymphomas, but *FOXO1* expression was significantly higher in lymphomas than in other cancer cell lines. These results indicates that *FOXO1* may play an important role in the pathogenesis of lymphoma and be an attractive target for lymphoma therapy. DLBC is the most common type of lymphoma worldwide, and patients with treatment failure after the chemoradiotherapy regimen of R-CHOP often have dismal outcomes with limited therapeutic options [47]. The importance of *FOXO1* in DLBC is underlined by the evidence that its mutation leads to competitive expansion of B cells during germinal center responses [48]. In the study, we found that the *FOXO1* gene was remarkably upregulated, while the *FOXO4* gene was decreased in DLBC, which belongs to B-cell lymphoma (Figure 2B). Of note, we found *FOXO4* expression was reduced only in B-cell lymphoma and not in other types of lymphoma (Figure 2A). Considering the importance of *FOXO1* and specificity of *FOXO4* in DLBC, we hypothesized that the combination of high expression of *FOXO1* with low expression of *FOXO4* could be diagnostic indicators for DLBC. Tamoxifen, an antiestrogen widely used in advanced ovarian and breast cancer has a therapeutic effect in DLBC by targeting estrogen receptor β [49]. In particular, we found the stimulation of Tamoxifen can reduce the expression of *FOXO1* and increase the expression of *FOXO4*. Accordingly, the clinical significance of tamoxifen in DLBC and its interaction with *FOXO1* and *FOXO4* deserved to be investigated.

In general, GBM is the most common and aggressive malignant primary brain tumor; thus, research on pathogenic mechanisms and new therapeutic modalities merits further attention. Evidences revealed that dysregulation of *FOX* genes contribute to aggressive tumor biology and therapy resistance in GBM patients [50]. Compared to normal tissues, *FOXM1* expressed at a higher level in GBM (Figure 3B). A circBFAR/miR−548b/*FOXM1* axis [51] and a *FOXM1*/ADAM17 feedback loop [52] have been identified as involved in the development of GBM. In this light, *FOXM1* might serve as a potential target for GBM. As can be seen in Figure 3A, *FOXO4* is decreased in several types of nervous system tumors, including GBM. It is reported that *FOXO4* possesses an anticancer glioma activity [53]. Therefore, molecular mechanism studies are warranted to clarify and assess whether elevation of *FOXM1* and simultaneously decrease of *FOXO4* could act as a diagnostic marker for GBM. Typically, LGG occurs in young adults. It has a high risk of turning malignant, which leads to neurological problems and ultimately death. Figure 3C,E clearly show that the expression of *FOXD1* is increased in LGG and its high expression predicts a poor prognosis of LGG patients, suggesting that *FOXD1* plays an important role in the pathogenesis of LGG. Then, we found a possible link between the high expression of *FOXD1* in LGG and the mutation of the tumor suppressor TP53 (Figure 3D). Since *FOXD1*-KD could potentiate the anticancer effects of radiotherapy by fostering the expression of TXNIP, an activator of TP53 in oral cancer cells, we speculate that *FOXD1* might have a similar role in LGG. Since MTX is the only anticancer drug that can inhibit the expression of *FOXD1* in Figure 8A, and there is evidence that MTX decreases the cells viability of glioma cells [54], further validation is required to clarify the role of MTX in this tumor.

In recent years, the incidence of hormone-dependent malignant tumors, led by breast cancer and prostate cancer, has been increasing year by year worldwide. *FOXA1*, an androgen receptor (AR) chromatin-binding precursor in the prostate epithelium, was reprogrammed to neuroendocrine-specific regulatory elements [55]. In addition, a previous study revealed that *FOXA1*, which established estrogen-responsive transcriptomes, was a pioneer of nuclear receptor action in breast cancer [56]. The interaction of *FOXA1* with co-factors such as ESR1 and E2F1 enhanced the susceptibility of breast cancer [57]. Clinically, combining gene therapy of *FOXA1* knockdown with ultrasound targeted microbubble destruction technology has been proved effective in the non-invasive therapy of breast cancer [58]. Consistently, the importance of *FOXA1* in breast and prostate cancer is underlined by our results that *FOXA1* is highly expressed in various prostate and breast cancers, and that *FOXA1* expression at mRNA in prostate cancer is significantly higher than that in other cancer lines (Figure 4A,B and Figure 5A). As an aggressive and highly lethal disease, TNBC is characterized by the lack of hormone receptors (HR). Of note, the expression of FOXA1 is low in TNBC but high in HR + breast cancer patients (Figure 5I). Therefore, it is clear that FOXA1 is a therapeutic target for HR + breast cancer. More importantly, the chemotherapeutic drug Etoposide, which has been shown to inhibit the growth of prostate and breast cancer [59,60], reduced FOXA1 expression in our study (Figure 8A), concluding that FOXA1 may be useful as potential prognostic marker and therapeutic target. All in all, our and others’ studies provide clear evidence for the role of FOXA1 in breast cancer and prostate cancer progression and treatment response, suggesting that the targeting of FOXA1 may be useful, in combination with standard therapy, in treatment.

It is noteworthy that TNBC and basal-like breast cancer (BLBC) are different molecular classes of breast cancer with a high degree of overlap, and the overlap ratio of gene expression profile between TNBC and BLBC can be as high as 80% [61]. Thus, we compared and analyzed the data from Figure 5A,I; the expression trend of *FOXC1*, *FOXK2*, and *FOXM1* in TNBC was indeed consistent with that in BLBC. Simultaneous elevation of these genes may serve as oncogenic targets of TNBC. Combining the results of Figure 5I and Figure S2, the use of MTX led to the inhibition of *FOXC1*, *FOXK2*, and *FOXM1* expression, which was highly expressed in TNBC. Consistent with our results, MTX has a spectrum of antitumor activity and can be used in the treatment of breast cancer [62]. Beware of the inhibitory effect of Trichostatin A (TSA) on FOXC1 and FOXK2 expression; despite the present study confirming its tumor suppressor effect on TNBC cells [63], more research is needed to prove its actual roles in TNBC patients.

As the most prevalent type of renal cell carcinoma, KIRC is characterized by genetic mutations in factors governing the hypoxia signaling pathway [64]. Our study also found that the expression of *FOXO1* and *FOXO4* was significantly downregulated in KIRC, which was strongly correlated with worse survival outcomes (Figure 6B,C). Meanwhile, there is a positive relationship between the two genes (Figure 6D). Therefore, the combined effect of *FOXO1* and *FOXO4* could be developed as a promising therapeutic strategy of KIRC. Lung cancer is a lethal tumor, which could be split into two basic types—small cell lung cancer and non-small-cell lung cancer (NSCLC). Lung cancer is largely caused by NSCLCs, with LUAD and LUSC being the most common forms. As displayed in Figure 6E, compared with other *FOX* family genes, *FOXM1* is significantly higher in these two types of lung cancer. Similar to our results, it is proved that *FOXM1* could be served as a predictive factor for patients with NSCLC [14]. Moreover, *FOXM1* could also promote the metastasis of lung cancer cells through the activation of the AKT/p70S6K signal pathway [65]. These new insights may contribute to further investigations about the role of *FOXM1* in therapeutic response of lung cancer. Given that Fulvestrant can reduce FOXM1 expression (Figure 8A) and that the combination of Fulvestrant and TLR4-specific inhibitor CLI−095 prevents the metastasis of NSCLC effectively [66], it is of potential significance to investigate the role of FOXM1 in Fulvestrant against NSCLC.

PAAD is the most devastating type of cancer, with a five-year survival rate of 10% [67]. Patients’ advanced disease at diagnosis remains a major challenge in treatment. Reliable predictors for diagnosis and individual risk of progression of PAAD are not available. In this report, *FOXM1* were expressed at relatively higher levels in PAAD tissues (Figure 7A,B) and predicted poor prognosis in PAAD (Figure 7C). In line with our report, *FOXM1* upregulation is critical in the initiation, advancement, and diversion of pancreatic cancer [68]. In Figure 7D,E, we paid attention to the role of potential oncogenic factor *FOXP1* in PAAD and assessed the possibility of *FOXP1* expression with cancer grade and disease progression. Zhao’s article demonstrated a novel TTN-AS1/microRNA−589−5p/*FOXP1* feedback loop in PAAD malignant phenotype [69], providing evidence for our results. In summary, these results implied a significant direction for further investigation of *FOXM1* and *FOXP1* during PAAD progression. As can be seen in Figure 8A, the treatment of Resveratrol lead to a decrease expression both in *FOXM1* and *FOXP1*. Moreover, in pancreatic cancer stem cells, Resveratrol can reverse epithelial-mesenchymal transition directly [70]. This discovery may result in novel targeted therapies after detailed mechanistic studies.

## 5. Conclusions

In this study, we revealed the differential expression of 43 *FOX* genes in 63 types of cancer diseases, and the responses of the *FOX* family to 60 chemical substances. On the one hand, based on the intersection of *FOX* genes that are differentially expressed in more than 30 cancers and respond to more than 30 chemotherapeutics simultaneously, the current study has identified six *FOX* genes, such as *FOXJ3*, *FOXK2*, *FOXN2*, *FOXN3*, *FOXO1*, and *FOXO3*, which play an important role in the development of a wide range of cancers and may share similar pathogenic mechanisms. On the other hand, some *FOX* genes were specifically expressed in certain cancer types; for instance, *FOXI3* is highly expressed only in GBM. The specific expression makes them excellent targets to cure cancers. More importantly, based on the differential expression of some *FOX* genes combination in a specific cancer and the response to related chemotherapeutic drugs, this study speculated the possibility of the combination of several *FOX* genes in the diagnosis and treatment of the certain tumors and the new application of old chemical substances. In addition, a detailed functional analysis of more *FOX* genes using mutants or overexpressing transgenic lines is required. Overall, our study provides clues for further functional analysis of *FOX* genes in the occurrence and therapy of cancers, which will help to open up new avenues for cancer prevention, diagnosis, and treatment.

## Data Availability

Publicly available datasets were analyzed in this study. This data can be found here: [https://www.ebi.ac.uk/gxa; http://gepia2.cancer-pku.cn/#index; http://ualcan.path.uab.edu/index.html; https://www.proteinatlas.org/; https://string-db.org/; https://portals.broadinstitute.org/ccle/data; https://portal.gdc.cancer.gov/].

## Supplementary Materials

The following supporting information can be downloaded at: https://www.mdpi.com/article/10.3390/genes13101754/s1, Figure S1: Differential expression of *FOX* genes in different cancer diseases. Figure S2: FOX genes were expressed in response to 60 different chemical sub-stances.

## References

[1] Kaestner K.H., Knochel W., Martinez D.E. (2000). Unified nomenclature for the winged helix/forkhead transcription factors. Genes Dev..

[2] Lam E.W.F., Brosens J.J., Gomes A.R., Koo C.-Y. (2013). Forkhead box proteins: Tuning forks for transcriptional harmony. Nat. Rev. Cancer.

[3] Golson M.L., Kaestner K.H. (2016). Fox transcription factors: From development to disease. Development.

[4] Benayoun B.A., Caburet S., Veitia R.A. (2011). Forkhead transcription factors: Key players in health and disease. Trends Genet..

[5] Zhu H. (2016). Forkhead box transcription factors in embryonic heart development and congenital heart disease. Life Sci..

[6] Katoh M., Igarashi M., Fukuda H., Nakagama H., Katoh M. (2013). Cancer genetics and genomics of human FOX family genes. Cancer Lett..

[7] de Brachène A.C., Demoulin J.-B. (2016). FOXO transcription factors in cancer development and therapy. Cell Mol. Life Sci..

[8] Kapushesky M., Emam I., Holloway E., Kurnosov P., Zorin A., Malone J., Rustici G., Williams E., Parkinson H., Brazma A. (2010). Gene expression atlas at the European bioinformatics institute. Nucleic Acids Res..

[9] Tang Z., Kang B., Li C., Chen T., Zhang Z. (2019). GEPIA2: An enhanced web server for large-scale expression profiling and interactive analysis. Nucleic Acids Res..

[10] Chandrashekar D.S., Bashel B., Balasubramanya S.A.H., Creighton C.J., Ponce-Rodriguez I., Chakravarthi B., Varambally S. (2017). UALCAN: A Portal for Facilitating Tumor Subgroup Gene Expression and Survival Analyses. Neoplasia.

[11] Colwill K., Gräslund S. (2011). A roadmap to generate renewable protein binders to the human proteome. Nat. Methods.

[12] Barretina J., Caponigro G., Stransky N., Venkatesan K., Margolin A.A., Kim S., Wilson C.J., Lehár J., Kryukov G.V., Sonkin D. (2012). The Cancer Cell Line Encyclopedia enables predictive modelling of anticancer drug sensitivity. Nature.

[13] von Mering C., Huynen M., Jaeggi D., Schmidt S., Bork P., Snel B. (2003). STRING: A database of predicted functional associations between proteins. Nucleic Acids Res..

[14] Tomczak K., Czerwińska P., Wiznerowicz M. (2015). The Cancer Genome Atlas (TCGA): An immeasurable source of knowledge. Contemp. Oncol..

[15] Jiang R., Zhang B., Teng X., Hu P., Xu S., Zheng Z., Liu R., Tang T., Ye F. (2020). Validating a targeted next-generation sequencing assay and profiling somatic variants in Chinese non-small cell lung cancer patients. Sci. Rep..

[16] Zhu H. (2014). Targeting forkhead box transcription factors FOXM1 and FOXO in leukemia (Review). Oncol. Rep..

[17] Widick P., Winer E.S. (2016). Leukocytosis and Leukemia. Prim Care.

[18] Rose-Inman H., Kuehl D. (2017). Acute Leukemia. Hematol. Oncol. Clin. N. Am..

[19] Hallek M. (2017). Chronic lymphocytic leukemia: 2017 update on diagnosis, risk stratification, and treatment. Am. J. Hematol..

[20] Bennour A., Saad A., Sennana H. (2016). Chronic myeloid leukemia: Relevance of cytogenetic and molecular assays. Crit. Rev. Oncol. Hematol..

[21] Armitage J.O., Gascoyne R.D., Lunning M.A., Cavalli F. (2017). Non-Hodgkin lymphoma. Lancet.

[22] Jiang M., Bennani N.N., Feldman A.L. (2017). Lymphoma classification update: T-cell lymphomas, Hodgkin lymphomas, and histiocytic/dendritic cell neoplasms. Expert Rev. Hematol..

[23] DeWitt J.C., Mock A., Louis D.N. (2017). The 2016 WHO classification of central nervous system tumors: What neurologists need to know. Curr. Opin. Neurol..

[24] Good D.W., Stewart G.D., Hammer S., Scanlan P., Shu W., Phipps S., Reuben R., McNeill A.S. (2014). Elasticity as a biomarker for prostate cancer: A systematic review. BJU Int..

[25] Myatt S.S., Lam E.W.F. (2007). The emerging roles of forkhead box (Fox) proteins in cancer. Nat. Rev. Cancer.

[26] Barbieri C.E., Baca S.C., Lawrence M.S., Demichelis F., Blattner M., Theurillat J.-P., White T.A., Stojanov P., Van Allen E., Stransky N. (2012). Exome sequencing identifies recurrent SPOP, FOXA1 and MED12 mutations in prostate cancer. Nat. Genet..

[27] Jin H.-J., Zhao J.C., Ogden I., Bergan R.C., Yu J. (2013). Androgen receptor-independent function of FoxA1 in prostate cancer metastasis. Cancer Res..

[28] Jiang X., Chen Y., Du E., Yang K., Zhang Z., Qi S., Xu Y. (2016). GATA3-driven expression of miR-503 inhibits prostate cancer progression by repressing ZNF217 expression. Cell. Signal..

[29] Shafer M.E.R., Nguyen A.H.T., Tremblay M., Viala S., Béland M., Bertos N.R., Park M., Bouchard M. (2017). Lineage Specification from Prostate Progenitor Cells Requires Gata3-Dependent Mitotic Spindle Orientation. Stem Cell Rep..

[30] Yang M., Li J.C., Tao C., Wu S., Liu B., Shu Q., Li B., Zhu R. (2021). PAQR6 Upregulation Is Associated with AR Signaling and Unfavorite Prognosis in Prostate Cancers. Biomolecules.

[31] Anastasiadi Z., Lianos G.D., Ignatiadou E., Harissis H.V., Mitsis M. (2017). Breast cancer in young women: An overview. Updat. Surg..

[32] Merino Bonilla J.A., Torres Tabanera M., Ros Mendoza L.H. (2017). Breast cancer in the 21st century: From early detection to new therapies. Radiologia.

[33] Jiang Y., Zou L., Lu W.-Q., Zhang Y., Shen A.-G. (2013). Foxo3a expression is a prognostic marker in breast cancer. PLoS ONE.

[34] Arruabarrena-Aristorena A., Maag J.L.V., Kittane S., Cai Y., Karthaus W.R., Ladewig E., Park J., Kannan S., Ferrando L., Cocco E. (2020). FOXA1 Mutations Reveal Distinct Chromatin Profiles and Influence Therapeutic Response in Breast Cancer. Cancer Cell.

[35] Bell R., Barraclough R., Vasieva O. (2017). Gene Expression Meta-Analysis of Potential Metastatic Breast Cancer Markers. Curr. Mol. Med..

[36] Tung N.M., Garber J.E. (2018). BRCA1/2 testing: Therapeutic implications for breast cancer management. Br. J. Cancer.

[37] Vagia E., Mahalingam D., Cristofanilli M. (2020). The Landscape of Targeted Therapies in TNBC. Cancers.

[38] Sung H., Ferlay J., Siegel R.L., Laversanne M., Soerjomataram I., Jemal A., Bray F. (2021). Global Cancer Statistics 2020: GLOBOCAN Estimates of Incidence and Mortality Worldwide for 36 Cancers in 185 Countries. CA Cancer J. Clin..

[39] Jia Z., Wan F., Zhu Y., Shi G., Zhang H., Dai B., Ye D. (2018). Forkhead-box series expression network is associated with outcome of clear-cell renal cell carcinoma. Oncol. Lett..

[40] Nasim F., Sabath B.F., Eapen G.A. (2019). Lung Cancer. Med. Clin. N. Am..

[41] Pierrou S., Hellqvist M., Samuelsson L., Enerbäck S., Carlsson P. (1994). Cloning and characterization of seven human forkhead proteins: Binding site specificity and DNA bending. EMBO J..

[42] Zhang J., Wang Y., Mo W., Zhang R., Li Y. (2020). The clinical and prognostic significance of FOXN3 downregulation in acute myeloid leukaemia. Int. J. Lab. Hematol..

[43] Long J., Jia M.Y., Fang W.Y., Chen X.J., Mu L.L., Wang Z.Y., Shen Y., Xiang R.F., Wang L.N., Wang L. (2020). FLT3 inhibition upregulates HDAC8 via FOXO to inactivate p53 and promote maintenance of FLT3-ITD+ acute myeloid leukemia. Blood.

[44] Miyazato P., Matsuoka M. (2014). Human T-cell leukemia virus type 1 and Foxp3 expression: Viral strategy in vivo. Int. Immunol..

[45] Zhao N., Pei S.N., Qi J., Zeng Z., Iyer S.P., Lin P., Tung C.H., Zu Y. (2015). Oligonucleotide aptamer-drug conjugates for targeted therapy of acute myeloid leukemia. Biomaterials.

[46] Gehringer F., Weissinger S.E., Swier L.J., Möller P., Wirth T., Ushmorov A. (2019). FOXO1 Confers Maintenance of the Dark Zone Proliferation and Survival Program and Can Be Pharmacologically Targeted in Burkitt Lymphoma. Cancers.

[47] Sehn L.H., Salles G. (2021). Diffuse Large B-Cell Lymphoma. N. Engl. J. Med..

[48] Roberto M.P., Varano G., Vinas-Castells R., Holmes A.B., Kumar R., Pasqualucci L., Farinha P., Scott D.W., Dominguez-Sola D. (2021). Mutations in the transcription factor FOXO1 mimic positive selection signals to promote germinal center B cell expansion and lymphomagenesis. Immunity.

[49] Langendonk M., de Jong M.R.W., Smit N., Seiler J., Reitsma B., Ammatuna E., Glaudemans A., van den Berg A., Huls G.A., Visser L. (2022). Identification of the estrogen receptor beta as a possible new tamoxifen-sensitive target in diffuse large B-cell lymphoma. Blood Cancer J..

[50] Robertson E., Perry C., Doherty R., Madhusudan S. (2015). Transcriptomic profiling of Forkhead box transcription factors in adult glioblastoma multiforme. Cancer Genom. Proteom..

[51] Li C., Guan X., Jing H., Xiao X., Jin H., Xiong J., Ai S., Wang Y., Su T., Sun G. (2022). Circular RNA circBFAR promotes glioblastoma progression by regulating a miR-548b/FoxM1 axis. FASEB J. Off. Publ. Fed. Am. Soc. Exp. Biol..

[52] Zhang C., Han X., Xu X., Zhou Z., Chen X., Tang Y., Cheng J., Moazzam N.F., Liu F., Xu J. (2018). FoxM1 drives ADAM17/EGFR activation loop to promote mesenchymal transition in glioblastoma. Cell Death Dis..

[53] Qi M., Sun L.A., Jiang X.C., Han Y.L., Wang L., Niu W.H., Fei M.X., Zhaba W.D., Zheng L.R., Zhou M.L. (2020). FOXO4 expression associates with glioblastoma development and FOXO4 expression inhibits cell malignant phenotypes in vitro and in vivo. Life Sci..

[54] Lopes D.V., de Fraga Dias A., Silva L.F.L., Scholl J.N., Sévigny J., Battastini A.M.O., Figueiró F. (2021). Influence of NSAIDs and methotrexate on CD73 expression and glioma cell growth. Purinergic Signal..

[55] Baca S.C., Takeda D.Y., Seo J.H., Hwang J., Ku S.Y., Arafeh R., Arnoff T., Agarwal S., Bell C., O’Connor E. (2021). Reprogramming of the FOXA1 cistrome in treatment-emergent neuroendocrine prostate cancer. Nat. Commun..

[56] Seachrist D.D., Anstine L.J., Keri R.A. (2021). FOXA1: A Pioneer of Nuclear Receptor Action in Breast Cancer. Cancers.

[57] Wen W., Chen Z., Bao J., Long Q., Shu X.O., Zheng W., Guo X. (2021). Genetic variations of DNA bindings of FOXA1 and co-factors in breast cancer susceptibility. Nat. Commun..

[58] Zhao R., Liang X., Zhao B., Chen M., Liu R., Sun S., Yue X., Wang S. (2018). Ultrasound assisted gene and photodynamic synergistic therapy with multifunctional FOXA1-siRNA loaded porphyrin microbubbles for enhancing therapeutic efficacy for breast cancer. Biomaterials.

[59] Liu F., Zhang H., Sun Z., Meng X., Ma Z., Wang Z. (2022). Effects of etoposide combined with cisplatin on prognosis of patients with castration-resistant prostate cancer who failed castration treatment. Am. J. Transl. Res..

[60] Koushki M., Khedri A., Aberomand M., Akbari Baghbani K., Mohammadzadeh G. (2021). Synergistic anti-cancer effects of silibinin-etoposide combination against human breast carcinoma MCF-7 and MDA-MB-231 cell lines. Iran. J. Basic Med. Sci..

[61] Dogra A., Mehta A., Doval D.C. (2020). Are Basal-Like and Non-Basal-Like Triple-Negative Breast Cancers Really Different?. J. Oncol..

[62] Yang V., Gouveia M.J., Santos J., Koksch B., Amorim I., Gärtner F., Vale N. (2020). Breast cancer: Insights in disease and influence of drug methotrexate. RSC Med. Chem..

[63] Song X., Wu J.Q., Yu X.F., Yang X.S., Yang Y. (2018). Trichostatin A inhibits proliferation of triple negative breast cancer cells by inducing cell cycle arrest and apoptosis. Neoplasma.

[64] Wolf M.M., Kimryn Rathmell W., Beckermann K.E. (2020). Modeling clear cell renal cell carcinoma and therapeutic implications. Oncogene.

[65] Bychkov A., Saenko V., Nakashima M., Mitsutake N., Rogounovitch T., Nikitski A., Orim F., Yamashita S. (2013). Patterns of FOXE1 expression in papillary thyroid carcinoma by immunohistochemistry. Thyroid.

[66] Fan S., Liao Y., Qiu W., Li L., Li D., Cao X., Ai B. (2020). Targeting Toll-like receptor 4 with CLI-095 (TAK-242) enhances the antimetastatic effect of the estrogen receptor antagonist fulvestrant on non-small cell lung cancer. Clin. Transl. Oncol. Off. Publ. Fed. Span. Oncol. Soc. Natl. Cancer Inst. Mex..

[67] Tempero M.A., Malafa M.P., Al-Hawary M., Behrman S.W., Benson A.B., Cardin D.B., Chiorean E.G., Chung V., Czito B., Del Chiaro M. (2021). Pancreatic Adenocarcinoma, Version 2.2021, NCCN Clinical Practice Guidelines in Oncology. J. Natl. Compr. Cancer Netw. JNCCN.

[68] Huang C., Du J., Xie K. (2014). FOXM1 and its oncogenic signaling in pancreatic cancer pathogenesis. Biochim. Biophys. Acta.

[69] Zhao J., Wu F., Yang J. (2021). A novel long non-coding RNA TTN-AS1/microRNA-589-5p/FOXP1 positive feedback loop increases the proliferation, migration and invasion of pancreatic cancer cell lines. Oncol. Lett..

[70] Hoca M., Becer E., Kabadayı H., Yücecan S., Vatansever H.S. (2020). The Effect of Resveratrol and Quercetin on Epithelial-Mesenchymal Transition in Pancreatic Cancer Stem Cell. Nutr. Cancer.

