# Supplementary figures and images for: An Integrated Study on the Differential Expression of the FOX Gene Family in Cancer and Their Response to Chemotherapy Drugs
Source: Genes (Basel). 2022 Sep 28;13(10):1754. doi: 10.3390/genes13101754 (PMC9602029; doi:10.3390/genes13101754)

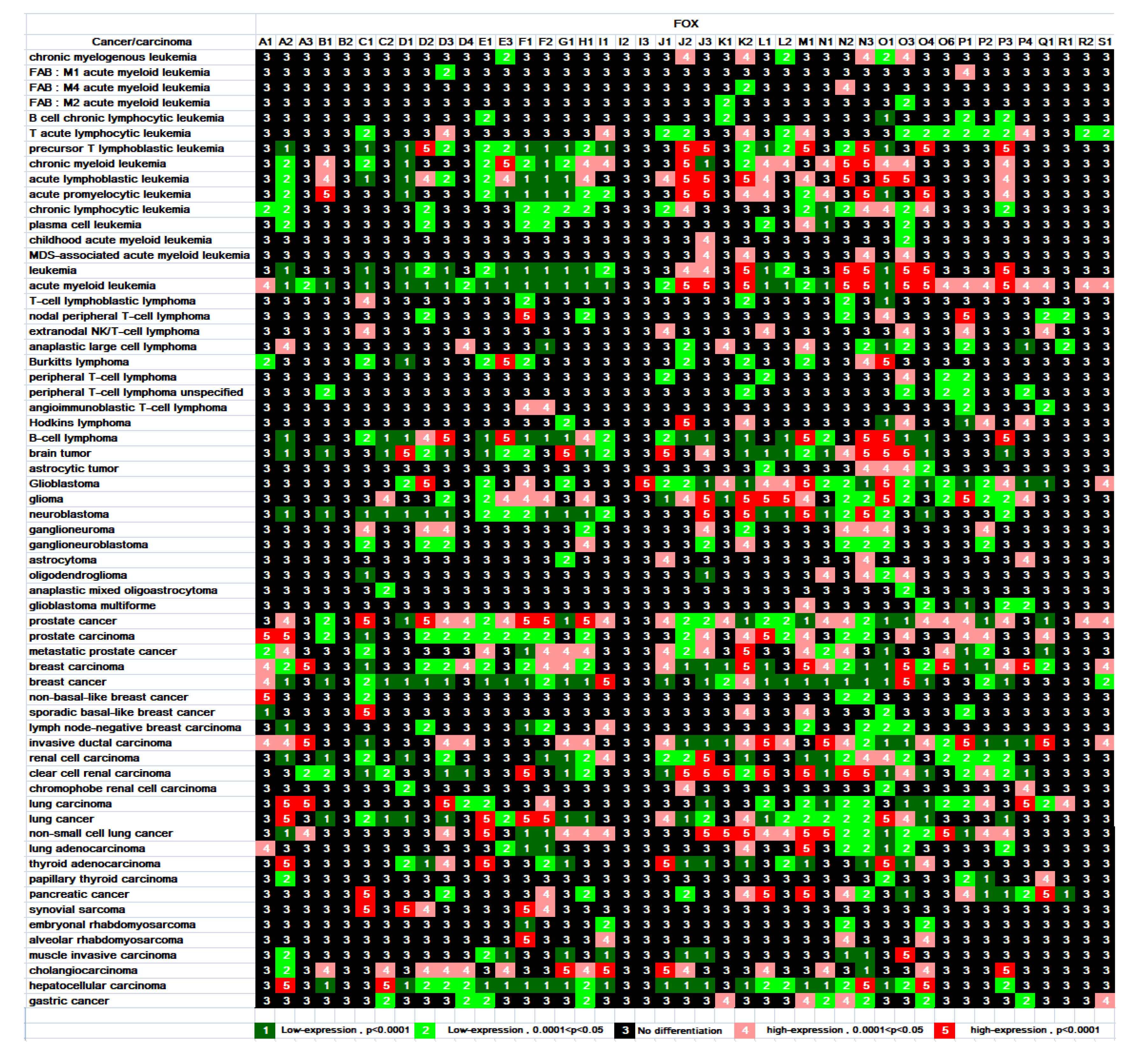

Supplement: Supplementary file 1 [file genes-13-01754-s001.zip › Figure S1.tif]

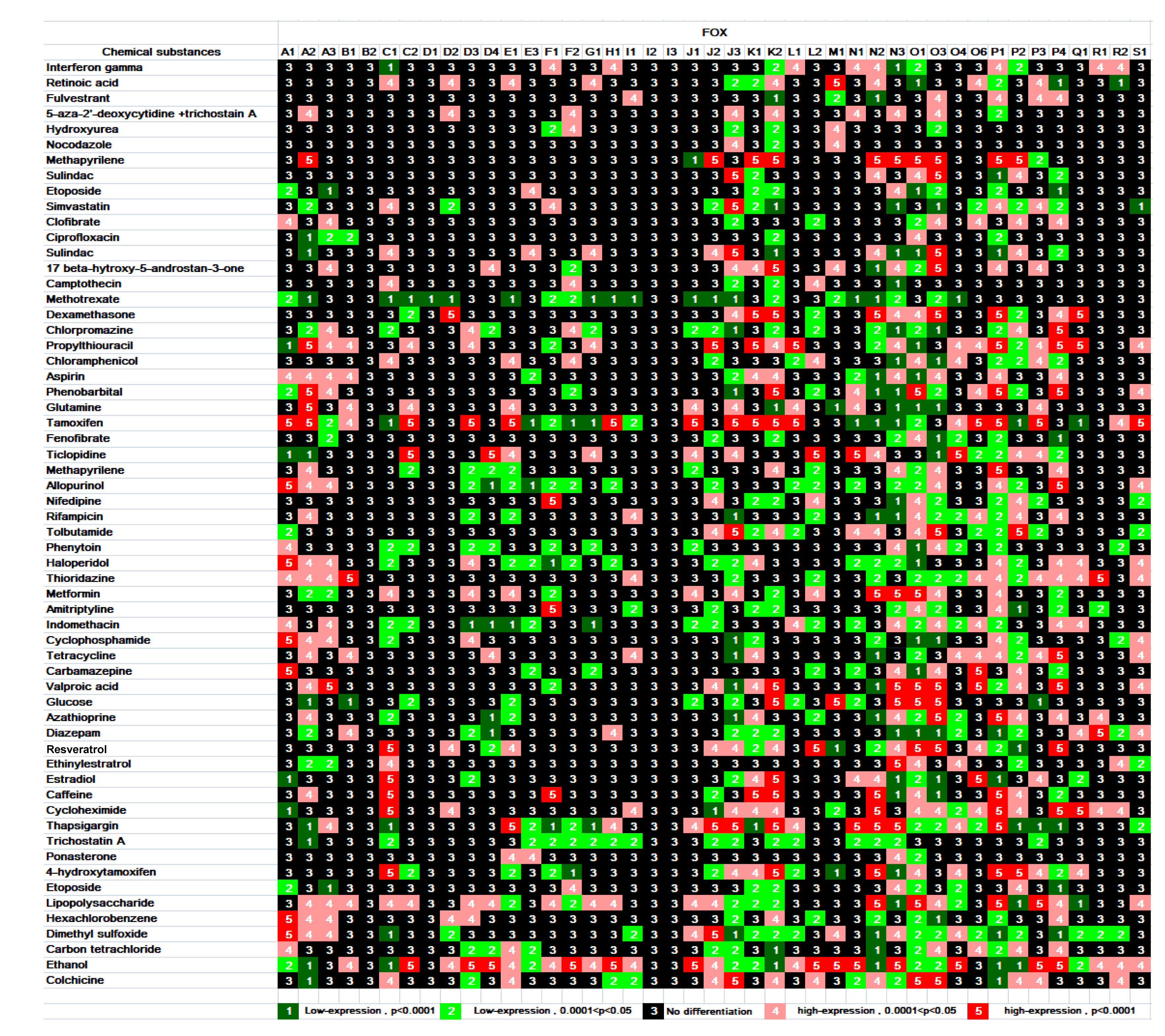

Supplement: Supplementary file 1 [file genes-13-01754-s001.zip › Figure S2.tif]
